# Harnessing the TP53INP1/TP53I3 axis for inhibition of colorectal cancer cell proliferation through MEG3 and Linc-ROR Co-expression

**DOI:** 10.1016/j.heliyon.2024.e34075

**Published:** 2024-07-10

**Authors:** Mahboobeh Ramezani, Fatemeh T. Shamsabadi, Majid Shahbazi

**Affiliations:** a Department of Genetics, School of Advanced Technologies in Medicine, Golestan University of Medical Sciences, Gorgan, Iran; b Department of Medical Biotechnology, School of Advanced Technologies in Medicine, Golestan University of Medical Sciences, Gorgan, Iran; c Medical Cellular & Molecular Research Center, Golestan University of Medical Sciences, Gorgan, Iran; d AryaTinaGene (ATG) Biopharmaceutical Company, Gorgan, Iran

**Keywords:** Colorectal cancer, MEG3, Linc-ROR shRNA, TP53

## Abstract

Dysregulation of long noncoding RNAs (lncRNAs), such as maternally expressed gene 3 (MEG3) and long intergenic noncoding RNA regulator of reprogramming (linc-ROR), plays a crucial role in colorectal cancer progression. We aimed to assess linc-ROR silencing and MEG3 activation on the colorectal cancer cell proliferation simultaneously; and explore the underlying mechanisms in the TP53-associated Pathway.

The MEG3 and linc-ROR shRNA were cloned under the bidirectional CEA promoter (UM1). Subsequently, additional vectors were constructed to express linc-ROR shRNA (UM2) and MEG3 (UM3). After transfecting colorectal cancer cell lines with these recombinant vectors, experiments on cell viability, apoptosis, and cell cycle analysis were conducted. Furthermore, TP53's transcriptional activity and associated genes were assessed using quantitative real-time polymerase chain reaction (qRT-PCR).

Interestingly, UM1 significantly inhibited the proliferation of both cell lines than UM2 and UM3. In response to UM1, TP53 transcript remarkably increased in HCT116 cells (10.46) than SW480 cells (6.16); which resulted in up-regulation of TP53INP1, TP53I3, GDF15, CCKN1A and BAX, and down-regulation of G1 cyclins (D1, E1). The rate of apoptosis increased in HCT116 (36.35 %) and SW480 (16.64 %) cells than control. Moreover, UM1-transfected HCT116 cells exhibited a notable arrest in the G0/G1 phase, accompanied by a reduction in the G2/M cell population.

Compared to unidirectional vectors, the concurrent targeting approach enhanced TP53 activation at the transcription level. The cell response to UM1 resulted in rapid upregulation of TP53, leading to inhibition of cell proliferation, increased apoptosis, and cell cycle arrest. These findings suggest that the synergistic effect of targeting both MEG3 and linc-ROR could serve as a promising therapeutic strategy for TP53-associated colon cancer.

## 1 Introduction

Colorectal cancer (CRC) poses a significant global public health concern due to its high mortality and morbidity rates [1,2]. Multiple genetic and epigenetic alterations characterize transitions from normal colonic mucosa to invasive adenocarcinomas [3]. Recent studies have shed light on the role of lncRNAs in cancer pathogenesis [4,5]. Dysregulation of lncRNAs has been implicated in carcinogenesis and tumor progression, primarily through their involvement in diverse cell signaling pathways [6,7]. Therefore, investigating lncRNAs holds promise for a better understanding of the regulatory mechanisms underlying tumor occurrence and development.

LncRNAs can be classified into four categories based on their modes of action: signal, decoy, guide, and scaffold [8]. Extensive evidence has demonstrated a dysregulated expression profile of regulatory lncRNAs across various cancer types, where they can exert both oncogenic or tumor suppressor activities [[9], [10], [11]]. For instance, in CRC, lncRNAs FENDRR [12], HOXB-AS3 [13] and MEG3 [14] have been shown to suppress the growth of CRC. Meanwhile, lncRNAs CYTOR [15], HOTAIR [16], and linc-ROR [17] promote CRC progression.

The maternally expressed gene 3 (MEG3) is a tumor suppressor lncRNA on human chromosome 14q32 [18]. Its expression has been observed to decrease in various malignancies, including cervical, prostate, bladder, and colon cancer [19,20]. Several researchers have reported that the down-regulation of MEG3 influences the proliferation and progression of cancer cells [20]. Furthermore, the anticancer function of MEG3 has been attributed to its ability to activate the p53 pathway, suggesting it is an underlying mechanism [21]. MEG3 has also been found to stimulate p53-dependent transcription and enhance protein stability in breast and gastric cancers [22,23].

The lincRNA-Regulator of reprogramming (linc-ROR) is a lncRNA located at chromosome 18q21.31 in humans and has been implicated as an oncogene in cancer [24]. It has been identified as an essential modulator in reprogramming differentiated cells to induce pluripotent stem cells [25]. Growing evidence has demonstrated its up-regulation in numerous malignant carcinomas, including breast, pancreatic, gallbladder, nasopharyngeal, and colon cancer [24,26,27]. Further research has revealed that linc‐ROR negatively regulates TP53 and inhibits p53‐mediated cell cycle progression and apoptosis [28,29]. In the context of CRC, linc-ROR has emerged as a potential and promising therapeutic target [26].

Given the emerging applications of MEG3 and linc-ROR in cancer biology, further access to their co-expression patterns is needed to better understand their regulatory roles and synergistic effects in CRC cells. Building upon previous reports, we hypothesized that MEG3 activation and linc-ROR silencing would influence TP53 transcriptionally and its associated genes for CRC treatment.

To investigate this, we designed and utilized a bidirectional carcinoma embryonic antigen promoter (CEA) to manipulate the expression of these lncRNAs. Additionally, we assessed the specific activity of the CEA promoter by comparing the expression level of these lncRNAs between colorectal cancer cells and HeLa cells.

## 2 Materials and methods

### 2.1 Plasmid construction

The sequences of MEG3 (NR_002766), linc-ROR (NR_048536.2), and the CEA promoter were retrieved from NCBI. To design the linc-ROR shRNA, InvivoGen's siRNA Wizard™ was then applied. In order to design a bidirectional construct, the sequences of MEG3 and linc-ROR shRNA were located in front of the 3′ site of the bidirectional CEA promoter (UM1) by the SnapGene software. Subsequently, the corresponding sequence was synthesized by the Biomatik Company (Canada) and substituted into the pRNA-U6.1/Neo vector. Also, the unidirectional vectors harboring only the shRNA-ROR (UM2) and the MEG3 (UM3) were constructed by removing another fragment using restriction enzyme digestion.

### 2.2 Cell culture and cell transfection

Human CRC cell lines, HCT116 and SW480, were obtained from the Pasteur Institute (Tehran,

IRAN). Cells were cultured in the DMEM-F12 medium (Bioidea, Cat. No.: BI-1012) supplemented with 10 % fetal bovine serum (Bioidea, Cat. No.: BI-1201) and 1 % penicillin/streptomycin antibiotic (Bioidea, Cat. No.: BI-1203). They were incubated at 37 °C in humidified air with 5 % CO2. The cells were transfected in the 6-well plates (0.75*106 cells/well) using Lipofectamine3000 (Invitrogen, Cat. No.: L3000001) according to the manufacturer's instructions [30].

Five experimental groups were designed including that (1) bidirectional vector containing the sequences of MEG3 and the linc-ROR shRNA (UM1), (2) unidirectional vector containing linc-ROR shRNA (UM2), (3) unidirectional vector containing the MEG3 sequence (4) empty vector, pRNAT-U6.1 (U6), and (5) blank cells (non-treated cells, NT) as control groups.

### 2.3 Cell viability assay

Cells were seeded into the 96-well plates and transfected with the UM1, UM2, and UM3 recombinant vectors and the U6 as a control. According to the manufacturer's instructions, the MTS solution (Promega, G3582) evaluated the viability of transfected cells at different time points [31]. The absorbance was measured at 490 nm using a microplate reader (Biotek, Germany).

### 2.4 Quantitative reverse transcription PCR (RT-qPCR)

The RNA was isolated from cells using RiboEx reagent (GeneAll, Cat. No.: 302-001) and converted to cDNA with a reverse transcription kit (Yekta Tajhiz Azma, Cat. No.: YT4500). Subsequently, the transcription levels of MEG3 and linc-ROR were evaluated by the SYBR PrimeScript RT-PCR kit (Takara, Cat. No.: RR066A). Moreover, the transcription level of TP53 and its associated genes including TP53INP1, TP53I3 (PIG3), GDF15, CDKN1A (P21), CCND1, CCNE1, and BAX were measured. Beta-actin was used as the internal control gene. The characteristics of primers are presented in Table 1.

**Table 1:** Primer sequences used in real-time PCR.

Primer	Sequence (5′–3′)
MEG3 forward	AGAACTGCGGATGGAAGC
MEG3 reverse	TGGCTGTGGAGGGATTTC
Linc-ROR forward	CAGCAGGTCTCAGGGTTG
Linc-ROR reverse	AGAGTGGCGATGTGTTTGG
TP53 forward	TGAGGTGCGTGTTTGTG
TP53 reverse	AGAGGAGCTGGTGTTGTTG
TP53I3 forward	GCAGAGACAAGGCCAGTATGAC
TP53I3 reverse	CAGTGACGTACTGAGCCTGG
TP53INP1 forward	TGACTTCATAGATACTTGCACTGG
TP53INP1 reverse	AGCAGGAATCACTTGTATCAGC
CDKN1A/P21 forward	CATGTGGACCTGTCACTGTCTTG
CDKN1A/P21 reverse	GGCGTTTGGAGTGGTAGAAATCTG
GDF15 forward	ACTCCGAAGACTCCAGATTCCG
GDF15 reverse	CGCACTTCTGGCGTGAGTATC
CCND1 forward	GTGGCCTCTAAGATGAAGGAGA
CCND1 reverse	TTGAGCTTGTTCACCAGGAGC
CCNE1 forward	GCCAGCCTTGGGACAATAATG
CCNE1 reverse	GAGCCTCTGGATGGTGCAATAATC
BAX forward	GCTTCAGGGTTTCATCCAG
BAX reverse	GTCCACGGCGGCAATCATC'
ACTB forward	AGCCTCGCCTTTGCCGA
ACTB reverse	GCGCGGCGATATCATCATC

### 2.5 Cell cycle and apoptosis assays

In order to assess the apoptotic effect of UM1 and its association in the cell cycle, the Annexin V-FITC/PI Kit (BioLegend, 640914) [32] and Propidium Iodide (Sigma,Cat.No.: P4170) [33] were applied. The staining process was carried out in colorectal cancer cells exposed to UM1 at 48 h as recommended by the manufacturer. Then, cells were analyzed using flow cytometry (Accuri™ C6 plus Cell). The percentage of cells in each cell cycle phase and apoptotic cells were quantified by the FlowJo data analysis and the FCS Express 7 software, respectively.

### 2.6 Evaluation of the specificity of CEA promoter

The specificity of the CEA promoter was evaluated by measuring the expression of MEG3 and linc-ROR shRNA in HCT116, SW480, and HeLa cancer cells that were transfected with the UM1.

### 2.7 Statistical analysis

Data analysis was performed using SPSS version 17.0 software (SPSS Inc., Chicago, IL). The difference of means was evaluated by the student's t-test or One-Way ANOVA. The values were presented as the mean ± SD. The differences were considered statistically significant when *p* ≤ 0.05.

## 3 Results

### 3.1 Construction of recombinant vectors

Three recombinant vectors were constructed and termed UM1 (8280 bp), UM2 (6679 bp), and UM3 (8216 bp). The map and characteristics of these recombinant vectors were illustrated using the SnapGene software (Fig. S1). The transcripts of MEG3 and linc-ROR shRNA are expressed under the control of the bidirectional CEA promoter. The resulting constructs were verified through double digestion assay and DNA sequencing.

### 3.2 UM1 vector impairs viability of HCT116 & SW480 cells

**Fig. 1:**
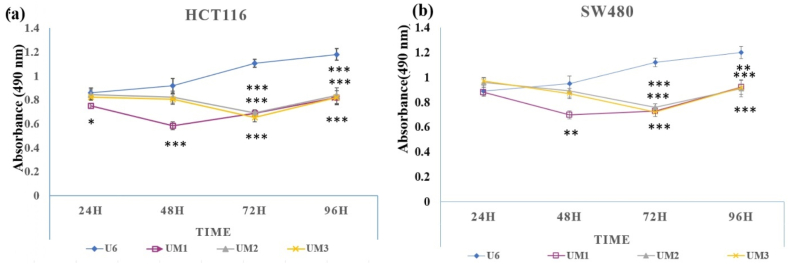
UM1 inhibits the proliferation rate of colorectal cancer cells. A significant reduction of cell viability was observed after 48 h post-transfection in HCT116 (a) and SW480 (b) cells. (***p < 0.001, **p < 0.01).

To investigate the impact of UM1, UM2, and UM3 on the proliferation of CRC cells, cell viability assay was carried out at 24, 48, 72, and 96 h post-transfection. The results revealed a significant decrease in the proliferative ability at 48 h in both cell lines treated with UM1 (*p* < 0.05) compared to the other groups (Fig. 1a and b). Conversely, in UM2-and UM3-treated groups, significant reductions in cell proliferation were observed at 72 h. Furthermore, it is apparent from the data that both cell lines show a similar response to UM1, UM2, and UM3. A comparison of cell proliferation between the two lines demonstrates lower viability in HCT116 cells.

### 3.3 Effect of UM1 on downstream TP53-associated genes

**Fig. 2:**
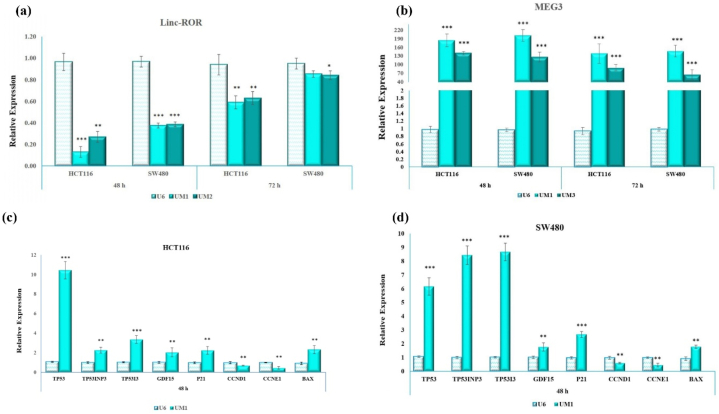
The relative expression of linc-ROR, MEG3, TP53, and its target genes in CRC cells. (a), **(b)** illustrate the expression of linc-ROR and MEG3 in CRC cells exposed to the UM1, UM2, and UM3. Fold-change expression of linc-ROR was significantly reduced at 48 h post-transfection **(a)**. Meanwhile, the expression of MEG3 remarkably increased after 48 h and 72 h post-transfection **(b)**. **(c), (d)** represent the expression of TP53, TP53INP1, TP53I3, GDF15, CDKN1A and BAX genes in HCT116 and SW480 after 48 h transfection with UM1. Apart from CCND1 and CCNE1, transcripts of other TP53-associated genes were remarkably increased in both cell lines. The highest transcript level in HCT116 cells belonged to TP53, while in SW480 cells, it was TP53I3.

The relative expression of linc-ROR and MEG3 was evaluated in a time- and cell-type-dependent manner. Following transfection with UM1 and UM2, linc-ROR mRNA expression was significantly down-regulated at 48 and 72 h post-transfection, respectively (*p* < 0.05; Fig. 2a). This figure reveals a trend of increasing linc-ROR mRNA levels over time, which may be attributed to its functional role. Additionally, HCT116 cells exhibited the lowest linc-ROR mRNA expression compared to SW480 cells.

Moreover, the expression of MEG3 transcript was remarkably increased in response to UM1 and UM3 at 48 and 72 h, respectively (*p* < 0.01; Fig. 2b). Notably, there was a significant difference in MEG3 expression between HCT116 and SW480 cells. Overall, UM1 exerted differential effects on HCT116 cells in these measures.

Further analysis was conducted to investigate the impact of UM1 on the expression of TP53 and its associated genes in CRC cells. As shown in Fig. 2c, the fold change in transcript expression of TP53 (10.43), TP53INP1 (2.26), TP53I3 (3.36), GDF15 (2.03), CDKN1A (2.24), and BAX (2.31) was significantly increased in HCT116 cells, respectively (*p* < 0.05). However, no significant change in transcript levels of CCND1 (0.69) and CCNE1 (0.44) was observed.

Interestingly, in SW480 cells exposed to UM1, a remarkable upregulation in the transcript levels of TP53 (6.16), TP53INP1 (8.48), TP53I3 (8.67), GDF15 (1.77), CDKN1A (2.67) and BAX (1.78) was observed, respectively (Fig. 2d). Conversely, there was a significant decrease in the transcript levels of CCND1 (0.59) and CCNE1 (0.45) (*p* < 0.05).

### 3.4 Combinatorial effect of UM1 on apoptosis and cell cycle

**Fig. 3:**
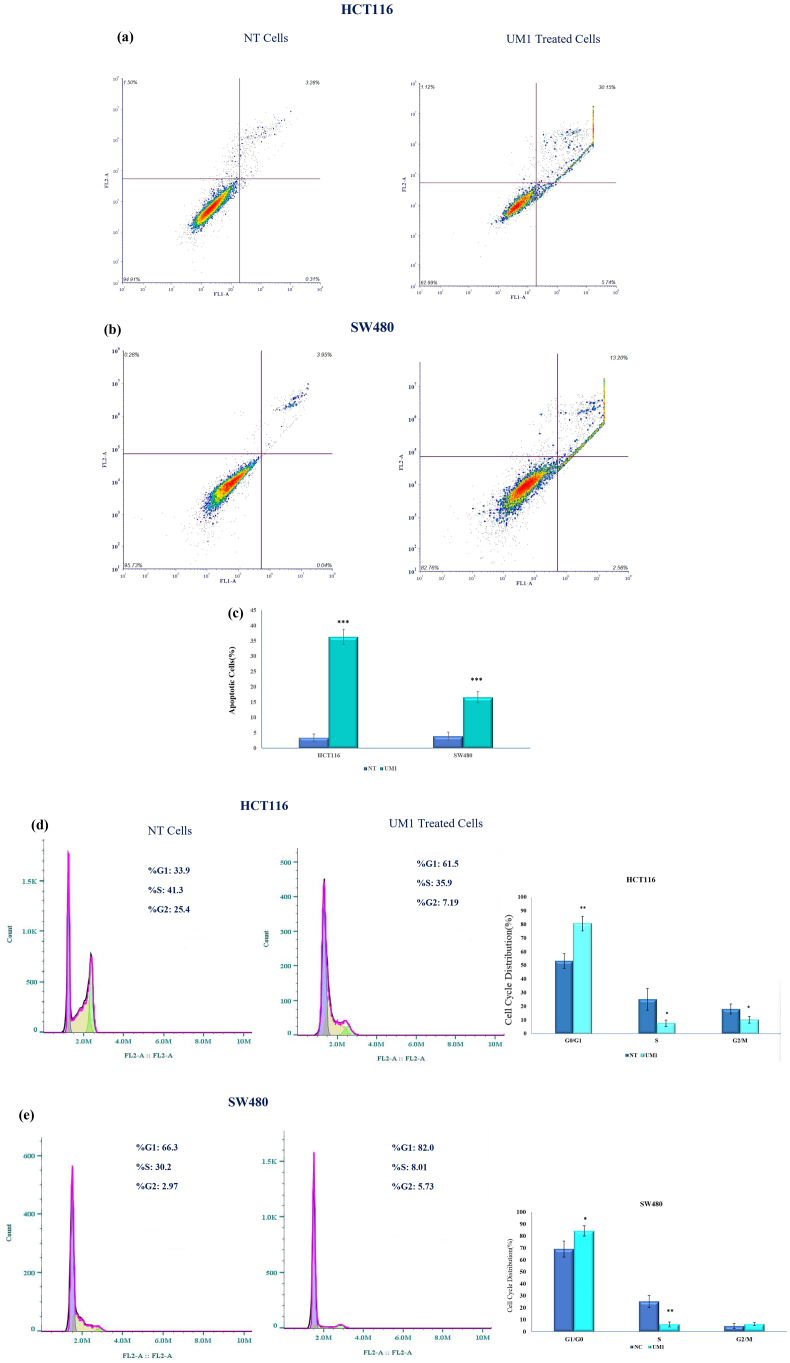
Apoptosis and cell cycle analysis of cells exposed to the UM1. Higher apoptotic cells were indicated in HCT116 cells **(a)** than in SW480 cells **(b)** in response to the activation of MEG3 and silencing of Linc-ROR. **(c)** The histogram chart shows the percentages of apoptotic cells in both cell lines. The G0/G1, S, or G2/M phase cell distribution was evaluated in HCT116 cells **(d)** and SW480 cells **(e)** across NT and UM1-transfected cells. The G0/G1 phase proportion significantly increased in both transfected cells compared to NT. Also, the percentage of the S phase was reduced remarkably (***p < 0.001, **p < 0.01, *p < 0.05).

Based on the initial statistical analysis, differences in cell viability regulated by UM1 were identified at 48 h post-transfection, attributed to the high expression of MEG3 and low expression of linc-ROR shRNA at this time point. Therefore, apoptosis ratios and cell cycle analysis were performed 48 h post-transfection. Fig. 3 shows the apoptotic rates in UM1-treated cells compared to the non-transfected (NT) cells. The apoptotic rates were 36.35 % and 16.64 % in HCT116 (Fig. 3a) and SW480 (Fig. 3b) cells in response to the UM1, respectively. On the other hand, the rate/population of apoptotic cells were significantly increased in both cell lines (Fig. 3c).

Moreover, cell cycle analysis was conducted by measuring DNA content in both transfected cell lines and the control. In response to UM1 in HCT116 cells (Fig. 3d) and SW480 cells (Fig. 3e), the cell population in the G0/G1 phase was significantly increased from 33.9 % (NT) to 61.5 % (*p* < 0.01) and from 66.3 % (NT) to 82 % (*p* < 0.01), respectively. Additionally, the distribution of the G2/M phase was altered from 25.4 % (NT) to 7.19 % in HCT116 cells. However, when SW480 cells were exposed to UM1, no significant difference in the number of cells in the G2/M phase was detected (*p* > 0.05). These data indicate that UM1 induces apoptosis and G0/G1 cell cycle arrest in both cell lines.

### 3.5 Colon-specific activity of CEA promoter

**Fig. 4:**
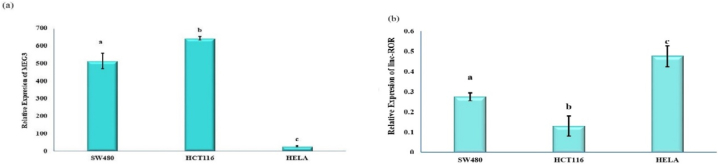
Colon-specific activity of CEA promoter. The values of MEG3 **(a)** and the linc-ROR **(b)** at the transcript level were significantly regulated by a bidirectional CEA promoter in colon cancer cell lines compared to HeLa cells.

The colon-specific activity of the CEA promoter was assessed by evaluating the expression of MEG3 and linc-ROR in HCT116, SW480, and HeLa cancer cells. Interestingly, a significant differential expression of MEG3 and linc-ROR was observed only in colorectal cancer cells (Fig. 4 a,b). These findings indicate that the bidirectional CEA promoter influences gene expression in colon cancer.

## 4 Discussion

Mounting evidence has demonstrated the crucial roles of lncRNAs in regulating different biological processes in cells. Dysregulation of lncRNAs has been associated with human diseases, including colon carcinoma. Some lncRNAs have been identified as potential biomarkers and therapeutic targets for diseases, including cancer [10,34]. In particular, several lncRNAs have been found to interact directly or indirectly with the tumor suppressor gene TP53, which plays a significant role in cancer initiation and progression. Targeting TP53-associated lncRNAs, such as MEG3 and linc-ROR, has emerged as a promising strategy for cancer treatment [22,35].

The present study aimed to investigate the impact of simultaneously targeting MEG3 and linc-ROR on the survival of HCT116 cells (p53+/+) and SW480 (p53+/−). The results showed that concurrent or single targeting of MEG3 and linc-ROR reduced cell proliferation. These findings are consistent with previous studies that reported the tumor suppressor function of MEG3 [36,37], downregulated in various tumor tissues and cells [35,38,39]. A possible explanation for this function might be that the MEG3 increases TP53 and GDF15 expression while reducing MDM2 expression at transcript and protein levels [21,40,41]. The session of MEG3 can suppress cell proliferation and inhibit tumor growth in different cancers [35,42,43].

On the other hand, linc-ROR has been identified as an oncogene that is upregulated in various types of cancer, exerting regulatory effects on proliferation, invasion, angiogenesis, and cancer stem cell pathways [[44], [45], [46]]. In our study, we observed the increased TP53 expression in cells exposed to UM1 and UM3, compared to single targeting. This upregulation of TP53 expression can be attributed to the reduction of linc-ROR, indicating a regulatory relationship between linc-ROR and TP53. Previous studies have identified linc-ROR as a negative regulator of TP53 translation, as it interacts with hnRNP I in MCF-7 and HCT116 cancer cells [29,47]. Other research groups have also shown that linc-ROR inhibits TP53 expression and affects p53-associated genes in colorectal cancer cell lines [26,29].

Moreover, the upregulation of linc-ROR expression at 72 h suggests the existence of a feedback loop between linc-ROR and p53 protein [48]. Furthermore, Linc-ROR's impact on p53 protein levels occurs following DNA damage, suggesting its involvement in stress response pathways. This post-transcriptional regulation of TP53 by linc-ROR influences downstream targets, including p21 and miR-145, and affects cellular processes such as G2/M cell cycle arrest and apoptosis [49].

Furthermore, MEG3 has been identified as a significant factor, if not the only one, in enhancing the stability and transcriptional activity of p53 in hepatoma cells [21]. Previous studies have reported that MEG3 activates p53-dependent pathways by downregulating MDM2, resulting in p53 stabilization and accumulation in the cytoplasm [22,50]. The stabilized form of p53 then transcriptionally activates downstream targets crucial in determining cell fate, mediating either apoptosis or cell cycle arrest [51].

The distinct impact of UM1 on cell viability, apoptosis, and cell cycle progression in HCT116 and SW480 cells is noteworthy. Our data demonstrate that UM1 induces a significantly higher apoptotic rate, approximately 36 %, in HCT116 cells compared to SW480 cells. Furthermore, UM1 induces G0/G1 cell cycle arrest in both cell lines, with a more pronounced effect observed in HCT116 cells. These findings are consistent with previous studies suggesting that overexpression of MEG3 and downregulation of linc-ROR promote cell apoptosis and G0/G1 arrest through p53 in colorectal cancer [18,23,43]. It has also been reported that these lncRNAs modulate p53-regulated processes, including cell cycle progression and apoptosis [21,50]. Therefore, it is reasonable to propose that the induction of apoptosis in these cells is associated with the upregulation of TP53.

Interestingly, we observed that UM1 effectively suppressed tumor cell proliferation, induced apoptosis, and caused cell cycle arrest in HCT116 cells more than in SW480 cells. Contrary to our expectations, we found a higher differential expression of p53-target genes in SW480 cells. While these effects are attributed to p53 activity and its downstream targets, another molecular mechanism may also be involved. Furthermore, our results suggest that the response to UM1 treatment may vary depending on the genetic background and molecular characteristics of different CRC cell lines.

Targeting the regulators that activate TP53/p53 and its target genes has been proposed as a therapeutic strategy [29,47,52]. In this study, we assessed the expression of p53-associated genes involved in apoptosis, cell cycle regulation, and cell growth processes in response to UM1 treatment. These findings shed light on the complex interplay between MEG3, linc-ROR, and TP53/p53 in colorectal cancer and highlight the potential of these lncRNAs as a therapeutic target in cancer treatment.

**Fig. 5:**
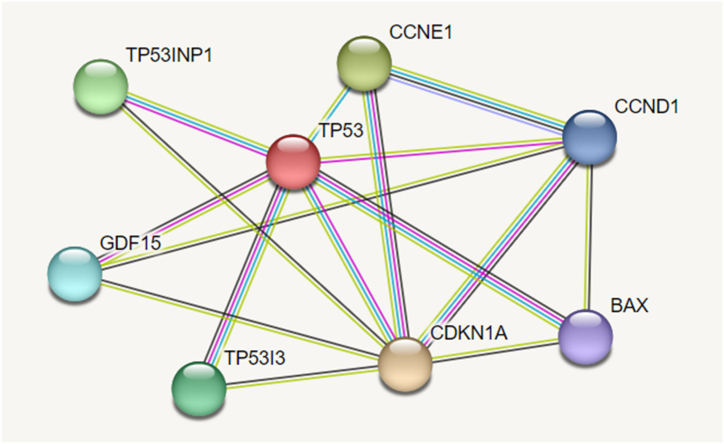
The protein-protein interactions of p53 and its associated genes on the STRING database. The physical interactions of p53 and its associated genes are selected with a medium score (0.400) according to the experimental (pink), co-expression (black), text mining (green), and curated database (cyan) criteria.

The up-regulation of TP53 and its associated genes, including TP53INP1, TP53I3, BAX, CDKN1A, and GDF15, in response to UM1 treatment, indicate the involvement of the p53 pathway in mediating the anti-proliferative effects of UM1 in CRC cells. This observation is supported by the network of protein-protein interactions revealed in the STRING database, which experimentally demonstrates their association (Fig. 5).

Phosphorylation of p53 at Ser-15 and Ser-20 has been demonstrated to occur upon initial DNA damage, stimulating its binding to promoter regions of specific genes. Examples of such genes include the G1 arrest genes p21, p53R2, and MDM2 [53]. This implies that UM1 may trigger a cascade of events through the activation of p53, leading to the transcriptional regulation of genes involved in cell cycle arrest, DNA repair, and inhibition of cell proliferation. Activation of the p53 pathway plays a crucial role in cellular processes such as apoptosis, cell cycle regulation, autophagy, and senescence in response to stress [[54], [55], [56]]. Our results demonstrate the up-regulation of these genes in transfected cells, accompanied by an increase in the apoptotic rate. These findings are consistent with previous studies that propose TP53INP1 and TP53I3 as molecules involved in p53-dependent apoptosis [53], regulated by directly binding p53 to their respective promoters [53,57,58].

Among the target genes of p53, tumor protein 53-induced nuclear protein 1 (TP53INP1) and TP53I3 hold significant importance. TP53INP1 is expressed in various tissues when exposed to stress agents. It acts as an anti-proliferation and pro-apoptosis protein, pivotal in p53-mediated cell cycle arrest and apoptosis across different cell types [59]. However, TP53INP1 is often down-regulated in different cancers, including CRC [60].

Studies have demonstrated that TP53INP1 enhances p53 protein stability and transcriptional activity by interacting with protein kinases HIPK2^1^ and protein kinase C. It activates p53 by regulating the phosphorylation ser-46, leading to apoptosis and cell growth arrest [53]. The modified version of p53 can activate the transcription of apoptosis-inducing genes and promote cell apoptosis [61,62].

The expression of TP53INP1 in cancer is attributed to the direct binding of p53 to the TP53INP1 promoter, as observed in various cancer types [63]. Restoring TP53INP1 expression could inhibit tumor growth through its anti-proliferative, pro-apoptotic, pro-autophagic, and anti-cell migration activities. TP53INP1 plays a crucial role in p53-dependent apoptosis. In association with HIPK2, it regulates p53 transcriptional activity on p21, PIG3, and BAX promoters, leading to G1 cell cycle arrest and increased p53-mediated apoptosis [53]. Furthermore, the upregulation of TP53INP1 expression has been linked to the pro-apoptotic role of MEG3, which acts as a miR-223 sponge and upregulates TP53 expression. The p53, a strictly regulated protein, plays a vital role in cell proliferation, cell cycle regulation, and cell apoptosis [64].

We observed increased PIG3 transcript upon UM1 treatment in both cell lines. This gene, along with Bax and CDKN1A (p21), participates in the p53 signaling pathway (hsa04115) [65]. Gene expression profile analysis revealed that the PIG3 transcript levels in HCT116 and SW480 cell lines are similar [66]. PIG3 is encoded through p53 binding to its promoter, which occurs before p53-induced apoptosis in colorectal cancer cells. Additionally, it has been shown that this protein stabilizes p53 by inhibiting its MDM2-mediated ubiquitination [67]. Moreover, the synergetic effect of high levels of p53 and PIG3 leads to the inhibition of catalase activity, resulting in the induction of apoptotic cell death [68]. These findings highlight the significance of the p53-PIG3 axis in mediating cellular responses and promoting apoptosis in CRC cells.

By expressing UM1, GDF15 was found to be highly transcribed in HCT116 cells (2.03) compared to SW480 cells (1.77). GDF15, a member of the TGF-β superfamily, is involved in multiple biological pathways, including the cytokine-cytokine receptor interaction pathway (hsa04060) and the colorectal cancer pathway (hsa05210) [65]. Comprehensive analysis of high throughput data has shown that GDF15 transcript is frequently expressed in various cancers, including colon adenocarcinoma [69]. Interestingly, the transcript level of GDF15 in HCT116 cells (10.20) was higher than in SW480 cells (9.24), highlighting the potential impact of GDF15 in the proliferation of colon cancer cells [66].

From an oncology perspective, a dual role has been proposed for GDF15, with its inhibition of carcinogenesis in normal tissues during the early stages of tumor development and its promotion of tumors at later stages. Indeed, its role in cancer can vary depending on the tumor type, disease stage, and microenvironment [70]. Molecular analysis has revealed that MEG3 has a p53-dependent function in enhancing the expression of GDF15 by binding to the GDF15 promoter while not binding to the p21Cip1 promoter [50].

This mechanism leads to the inhibition of proliferation in HCT116 cells. These findings suggest that MEG3 activates p53 and selectively promotes the expression of p53 target genes [20,52,71]. Interestingly, in addition to its p53-dependent role, MEG3 may also have p53-independent functions. The pivotal role of p53 in regulating cell proliferation is well-established [72]. Given the ability of MEG3 to selectively modulate its target genes by regulating TP53 levels, it is conceivable that MEG3 RNA interacts directly with p53, similar to apoptotic-stimulating proteins, leading to the stabilization of p53. These interactions influence p53's binding preferences to the promoters of BAX and PIG3. The differential response observed between the GDF15 and p21CIP1 promoters to MEG3 suggests the GDF15 promoter may exhibit higher sensitivity to lower levels of p53 compared to the p21CIP1 promoter [50]. This indicates that MEG3 has the potential to selectively regulate its target genes by modulating p53 levels.

Furthermore, MEG3 RNA can bind to the promoter region of its target gene, p21, accumulating p53. However, in HCT116 cells, no correlation was observed between MEG3 expression and the level of p21 [41].

The BAX gene, which is transcriptionally regulated by p53 and possesses pro-apoptotic activity, plays a crucial role in the colorectal cancer pathway (hsa05210) [65]. Gene expression profiling interactive analysis of BAX in colon adenocarcinoma has revealed a significantly higher expression of BAX compared to normal tissue [73]. Dimerization of BCL2 with BAX inhibits p53-mediated apoptosis, and the balance between apoptosis and survival is determined by the ratio of BCL2 to BAX [74]. MEG3 has been implicated in upregulating the expression of BAX and caspase 3, as demonstrated in prostate cancer [75]. Recent studies have reported the involvement of MEG3 in modulating various signaling pathways, including the PI3K/AKT/Bcl-2/Bax/Cyclin D1/p53 and PI3K/AKT/Bcl-2/Bax/P21 to suppress tumor growth in cervical cancer and pancreatic cancer, respectively [76]. Moreover, MEG3 has been shown to induce G0/G1 cell cycle arrest and enhance the expression of PTEN, BAX, and p53 protein in hepatocellular carcinoma [39].

The observed down-regulation of CCND1 and CCNE1, two genes involved in cell cycle progression, further supports the role of UM1 in inhibiting cell proliferation and promoting cell cycle arrest.

While our findings demonstrate the efficacy of UM1 treatment in reducing cell proliferation and inducing apoptosis in CRC cell lines, several limitations should be acknowledged, including utilizing a limited number of cell lines, relying on *in vitro* models, and the short-term assessment of treatment effects. Future studies should address these limitations by employing a broader range of cell lines, incorporating *in vivo* models to mimic the complex tumor microenvironment, and conducting long-term studies to assess sustained therapeutic responses and potential adaptive mechanisms. Moreover, based on our findings, we have proposed a plausible mechanism involving MEG3 and linc-ROR in cells treated with UM1 compared to NT cells. These results provide valuable insights into the intricate regulatory mechanisms governed by MEG3 and TP53, emphasizing the selective role of MEG3 in modulating p53-dependent gene expression.

**Fig. 6:**
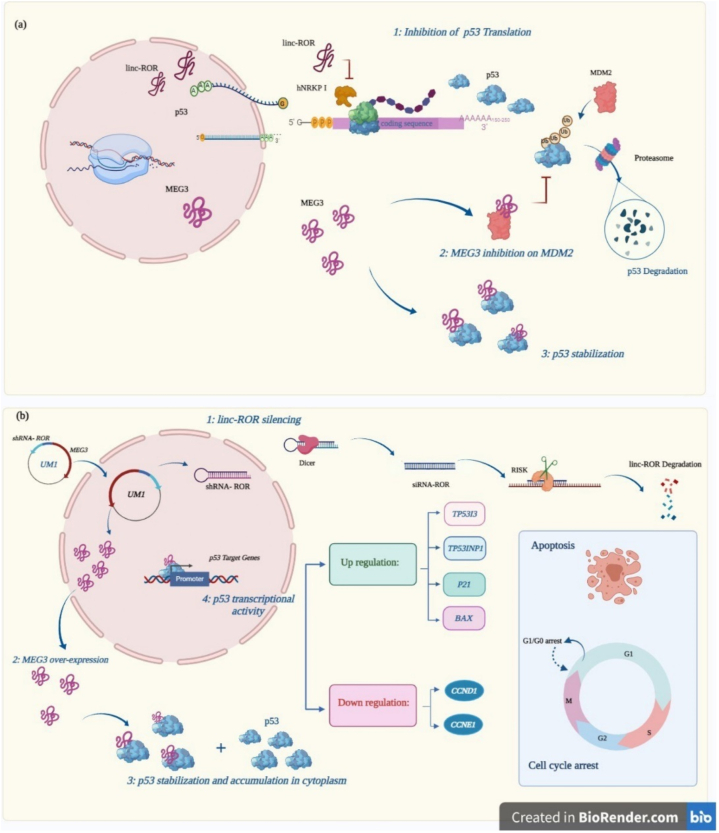
Schematic overview of MEG3 and linc-ROR functions in colon cancer and normal cells. (a) Function of MEG3 and linc-ROR in normal cells. The linc-ROR inhibits the TP53 translation by targeting hnRNPI. Meanwhile, the MEG3 reduces p53 ubiquitination and increments p53 instability by double targeting p53 and MDM2, respectively. (b) Expected function of UM1 in the HCT116 cells. The UM1 silences the linc-ROR and increases the MEG3 transcript, which increases p53 instability and accumulation in the cytoplasm. Also, the complex MEG3 and p53 activate the p53 target genes. These events lead to apoptosis and cell cycle arrest in the treated cells. This figure was created by BioRender (biorender.com).

Our molecular findings illustrate an expected mechanism of MEG3 and linc-ROR in cells introduced with the UM1 than NT cells (Fig. 6 a,b).

We also investigated the tissue-specific activity of the CEA promoter as a targeted approach in cancer gene therapy. We utilized a bidirectional vector, UM1, to assess its function in the HeLa cell line. The differential expression of MEG3 and linc-ROR between colorectal cancer cells and HeLa cells indicates that the bidirectional CEA promoter exerts a specific regulatory effect on gene expression in colon cancer, establishing it as a promising tumor-specific promoter [77]. These findings support earlier research [78,79] and further validate our recent bidirectional promoter design [80,81] trends. Our current results highlight the potential of utilizing the CEA promoter for targeted gene expression in CRC research and therapeutic interventions.

## 5 Conclusion

Overall, our findings emphasize the significance of simultaneously targeting TP53-associated lncRNAs, specifically MEG3, and linc-ROR, to modulate cell proliferation and enhance cell survival in CRC. These lncRNAs play crucial roles in cancer initiation and progression, making them promising therapeutic targets for cancer gene therapy. The results demonstrated that combined targeting of these lncRNAs effectively reduced cell proliferation compared to single targeting. Upon targeting MEG3 and linc-ROR, we observed the upregulation of TP53 and its target genes, including TP53INP1 and TP53I3, leading to apoptosis and cell cycle arrest. These findings underscore the therapeutic potential of targeting TP53-associated lncRNAs in cancer therapy and contribute to our growing understanding of the synergistic effect of these lncRNAs in cancer-specific treatment. By manipulating MEG3 and linc-ROR expression, we can effectively modulate the p53 pathway (hsa04115), inhibiting cancer cell growth and promoting programmed cell death, apoptosis.

## Funding

This work was financially supported by the 10.13039/501100008091Golestan University of Medical Sciences (Grant number 110114) and the AryaTinaGene (ATG) Biopharmaceutical Company.

## Ethics approval

The research was approved by the Ethics Committee of Golestan University of Medical Sciences, (code: IR.GOUMS.REC.1397.088).

## Data availability statement

The datasets used and analyzed in the present study are available from the corresponding author upon reasonable request.

## CRediT authorship contribution statement

**Mahboobeh Ramezani:** Writing – original draft, Investigation, Formal analysis. **Fatemeh T. Shamsabadi:** Writing – review & editing, Validation, Project administration, Formal analysis. **Majid Shahbazi:** Writing – review & editing, Validation, Supervision, Funding acquisition.

## Declaration of competing interest

The authors declare that they have no known competing financial interests or personal relationships that could have appeared to influence the work reported in this paper.

## Footnotes

1 Homeodomain-interacting protein kinase 2.

## References

[1] Bray F., Ferlay J., Soerjomataram I., Siegel R.L., Torre L.A., Jemal A. (2018). Global cancer statistics 2018: GLOBOCAN estimates of incidence and mortality worldwide for 36 cancers in 185 countries. CA. Cancer J. Clin..

[2] Lv L., Zhu W., Chen J., Gou X., Xu J., Zhu W., Zheng L., Shen X. (2021). Transcriptome analysis of FuZheng XiaoJi prescription inhibiting the proliferation of colorectal cancer. Life.

[3] Nguyen H.T., Duong H.Q. (2018). The molecular characteristics of colorectal cancer: implications for diagnosis and therapy. Oncol. Lett..

[4] Jiang M.C., Ni J.J., Cui W.Y., Wang B.Y., Zhuo W. (2019). Emerging roles of lncRNA in cancer and therapeutic opportunities. Am. J. Cancer Res..

[5] Ahadi A. (2021). Functional roles of lncRNAs in the pathogenesis and progression of cancer. Genes Dis.

[6] Taniue K., Akimitsu N. (2021). The functions and unique features of lncrnas in cancer development and tumorigenesis. Int. J. Mol. Sci..

[7] Huarte M. (2015). The emerging role of lncRNAs in cancer. Nat. Med..

[8] Gao N., Li Y., Li J., Gao Z., Yang Z., Li Y., Liu H., Fan T. (2020). Long non-coding RNAs: the regulatory mechanisms, research strategies, and future directions in cancers. Front. Oncol..

[9] Liao Z., Nie H., Wang Y., Luo J., Zhou J., Ou C. (2021). The emerging landscape of long non-coding RNAs in colorectal cancer metastasis. Front. Oncol..

[10] Slack F.J., Chinnaiyan A.M. (2019). The role of non-coding RNAs in oncology. Cell.

[11] Rao A.K.D.M., Rajkumar T., Mani S. (2017). Perspectives of long non-coding RNAs in cancer. Mol. Biol. Rep..

[12] Yang J., Hu Y., Tan Z., Zhang F., Huang W., Chen K. (2023). The lncRNA FENDRR inhibits colorectal cancer progression via interacting with and triggering GSTP1 ubiquitination by FBX8. Heliyon.

[13] Huang J.Z., Chen M., Chen D., Gao X.C., Zhu S., Huang H., Hu M., Zhu H., Yan G.R. (2017). A peptide encoded by a putative lncRNA HOXB-AS3 suppresses colon cancer growth. Mol. Cell.

[14] Zhu Y., Chen P., Gao Y., Ta N., Zhang Y., Cai J., Zhao Y., Liu S., Zheng J. (2018). MEG3 activated by vitamin D inhibits colorectal cancer cells proliferation and migration via regulating clusterin. EBioMedicine.

[15] Yue B., Liu C., Sun H., Liu M., Song C., Cui R., Qiu S., Zhong M. (2018). A positive feed-forward loop between LncRNA-CYTOR and wnt/β-catenin signaling promotes metastasis of colon cancer. Mol. Ther..

[16] Pan S., Liu Y., Liu Q., Xiao Y., Liu B., Ren X., Qi X., Zhou H., Zeng C., Jia L. (2019). HOTAIR/miR-326/FUT6 axis facilitates colorectal cancer progression through regulating fucosylation of CD44 via PI3K/AKT/mTOR pathway. Biochim. Biophys. Acta - Mol. Cell Res..

[17] Zhou P., Sun L., Liu D., Liu C., Sun L. (2016). Long non-coding RNA lincRNA-ROR promotes the progression of colon cancer and holds prognostic value by associating with miR-145. Pathol. Oncol. Res..

[18] Zheng Q., Lin Z., Xu J., Lu Y., Meng Q., Wang C., Yang Y., Xin X., Li X., Pu H., Gui X., Li T., Xiong W., Lu D. (2018). Long noncoding RNA MEG3 suppresses liver cancer cells growth through inhibiting β-catenin by activating PKM2 and inactivating PTEN article. Cell Death Dis..

[19] Wang W., Xie Y., Chen F., Liu X., Zhong L.L., Wang H.Q., Li Q.C. (2019). LncRNA MEG3 acts a biomarker and regulates cell functions by targeting ADAR1 in colorectal cancer. World J. Gastroenterol..

[20] Ghafouri-Fard S., Taheri M. (2019). Maternally expressed gene 3 (MEG3): a tumor suppressor long non coding RNA. Biomed. Pharmacother..

[21] Zhu J., Liu S., Ye F., Shen Y., Tie Y., Zhu J., Wei L., Jin Y., Fu H., Wu Y., Zheng X. (2015). Long noncoding RNA MEG3 interacts with p53 protein and regulates partial p53 target genes in hepatoma cells. PLoS One.

[22] Sun L., Li Y., Yang B. (2016). Downregulated long non-coding RNA MEG3 in breast cancer regulates proliferation, migration and invasion by depending on p53's transcriptional activity. Biochem. Biophys. Res. Commun..

[23] Yang X., Ren H., Wei R., Zhang X., Zhang X., Wang C., Zhang Y. (2016). MEG3 regulates cell cycle progression via control of P53 expression in gastric cancer. Int. J. Clin. Exp. Pathol..

[24] Wang L., Yu X., Zhang Z., Pang L., Xu J., Jiang J., Liang W., Chai Y., Hou J., Li F. (2017). Linc-ROR promotes esophageal squamous cell carcinoma progression through the derepression of SOX9. J. Exp. Clin. Cancer Res..

[25] Pan Y., Li C., Chen J., Zhang K., Chu X., Wang R., Chen L. (2016). The emerging roles of long noncoding RNA ROR (lincRNA-ROR) and its possible mechanisms in human cancers. Cell. Physiol. Biochem..

[26] Li H., Jiang X., Niu X. (2017). Long non-coding RNA reprogramming (ROR) promotes cell proliferation in colorectal cancer via affecting p53. Med. Sci. Monit..

[27] Gao S., Wang P., Hua Y., Xi H., Meng Z., Liu T., Chen Z., Liu L.M. (2016). ROR functions as a ceRNA to regulate Nanog expression by sponging miR-145 and predicts poor prognosis in pancreatic cancer. Oncotarget.

[28] Sun Z., Nie X., Sun S., Dong S., Yuan C., Li Y., Xiao B., Jie D., Liu Y. (2017). Long non-coding RNA MEG3 downregulation triggers human pulmonary artery smooth muscle cell proliferation and migration via the p53 signaling pathway. Cell. Physiol. Biochem..

[29] Zhang A., Zhou N., Huang J., Liu Q., Fukuda K., Ma D., Lu Z., Bai C., Watabe K., Mo Y.Y. (2013). The human long non-coding RNA-RoR is a p53 repressor in response to DNA damage. Cell Res..

[30] Schroeder K., Conrad G. (1983).

[31] coperation Promega (2001). http://www.promega.com.

[32] Gel U. (2012). http://www.biolegend.com.

[33] Zhu H. (2012). Propidium Iodide staining of cells for FACS analysis. Bio-Protocol.

[34] Singh D., Khan M.A., Siddique H.R. (2020). Emerging role of long non-coding RNAs in cancer chemoresistance: unravelling the multifaceted role and prospective therapeutic targeting. Mol. Biol. Rep..

[35] Wu X., Li J., Ren Y., Zuo Z., Ni S., Cai J. (2019). MEG3 can affect the proliferation and migration of colorectal cancer cells through regulating miR-376/PRKD1 axis. Am. J. Transl. Res..

[36] Zhou Y., Zhang X., Klibanski A. (2012). MEG3 noncoding RNA: a tumor suppressor. J. Mol. Endocrinol..

[37] Ma L., Wang F., Du C., Zhang Z., Guo H., Xie X., Gao H., Zhuang Y., Kornmann M., Goa H., Tian X., Yang Y. (2018). Long non-coding RNA MEG3 functions as a tumour suppressor and has prognostic predictive value in human pancreatic cancer. Oncol. Rep..

[38] Wang H., Li H., Zhang L., Yang D. (2018). Overexpression of MEG3 sensitizes colorectal cancer cells to oxaliplatin through regulation of miR-141/PDCD4 axis. Biomed. Pharmacother..

[39] Zhang Y., Liu J., Lv Y., Zhang C., Guo S. (2019). LncRNA meg3 suppresses hepatocellular carcinoma in vitro and vivo studies. Am. J. Transl. Res..

[40] He Y., Luo Y., Liang B., Ye L., Lu G., He W. (2017). Potential applications of MEG3 in cancer diagnosis and prognosis. Oncotarget.

[41] Lin T., Hou P.F., Meng S., Chen F., Jiang T., Le Li M., Shi M.L., Liu J.J., Zheng J.N., Bai J. (2019). Emerging roles of p53 related lncRNAs in cancer progression: a systematic review. Int. J. Biol. Sci..

[42] Zhang J., Yao T., Wang Y., Yu J., Liu Y., Lin Z. (2016). Long noncoding RNA MEG3 is downregulated in cervical cancer and affects cell proliferation and apoptosis by regulating miR-21. Cancer Biol. Ther..

[43] Physiology C. (2015).

[44] Hou L., Tu J., Cheng F., Yang H., Yu F., Wang M., Liu J., Fan J., Zhou G. (2018). Long noncoding RNA ROR promotes breast cancer by regulating the TGF-β pathway. Cancer Cell Int..

[45] Chen W., Yang J., Fang H., Li L., Sun J. (2020). Relevance function of linc-ROR in the pathogenesis of cancer. Front. Cell Dev. Biol..

[46] Gao H., Wang T., Zhang P., Shang M., Gao Z., Yang F., Liu R. (2020). Linc-ROR regulates apoptosis in esophageal squamous cell carcinoma via modulation of p53 ubiquitination by targeting miR-204-5p/MDM2. J. Cell. Physiol..

[47] Pal S., Garg M., Pandey A.K. (2020). Deciphering the mounting complexity of the p53 regulatory network in correlation to long non-coding RNAs (lncRNAs) in ovarian cancer. Cells.

[48] Su M., Wang H., Wang W., Wang Y., Ouyang L., Pan C., Xia L., Cao D., Liao Q. (2018). LncRNAs in DNA damage response and repair in cancer cells, Acta Biochim. Biophys. Sin. Biophys. Sin..

[49] Chaudhary R., Lal A. (2017).

[50] Zhou Y., Zhong Y., Wang Y., Zhang X., Batista D.L., Gejman R., Ansell P.J., Zhao J., Weng C., Klibanski A. (2007). Activation of p53 by MEG3 non-coding RNA. J. Biol. Chem..

[51] Kowalczyk A.E., Krazinski B.E., Godlewski J., Kiewisz J., Kwiatkowski P., Sliwinska-Jewsiewicka A., Kiezun J., Sulik M., Kmiec Z. (2017). Expression of the EP300, TP53 and BAX genes in colorectal cancer: correlations with clinicopathological parameters and survival. Oncol. Rep..

[52] Uroda T., Anastasakou E., Rossi A., Teulon J.M., Pellequer J.L., Annibale P., Pessey O., Inga A., Chillón I., Marcia M. (2019). Conserved pseudoknots in lncRNA MEG3 are essential for stimulation of the p53 pathway. Mol. Cell.

[53] Shahbazi J., Lock R., Liu T. (2013). Tumor protein 53-induced nuclear protein 1 enhances p53 function and represses tumorigenesis. Front. Genet..

[54] Hafner A., Bulyk M.L., Jambhekar A., Lahav G. (2019). The multiple mechanisms that regulate p53 activity and cell fate. Nat. Rev. Mol. Cell Biol..

[55] Lopes J.L., Chaudhry S., Lopes G.S., Levin N.K., Tainsky M.A. (2019). FANCM, RAD1, CHEK1 and TP53I3 act as BRCA-like tumor suppressors and are mutated in hereditary ovarian cancer. Cancer Genet.

[56] Zhang W., Luo J., Chen F., Yang F., Song W. (2015).

[57] Li M., Li S., Liu B., Gu M., Zou S., Xiao B., Yu L., Ding W. (2017). PIG3 promotes NSCLC cell mitotic progression and is associated with poor prognosis of NSCLC patients.

[58] Ito M., Nishiyama H., Watanabe J., Kawanishi H., Takahashi T., Kamoto T., Habuchi T., Ogawa O. (2006). Association of the PIG3 promoter polymorphism with invasive bladder cancer in a Japanese population. Jpn. J. Clin. Oncol..

[59] Tomasini R., Samir A.A., Carrier A., Isnardon D., Cecchinelli B., Soddu S., Malissen B., Dagorn J.C., Iovanna J.L., Dusetti N.J. (2003). TP53INP1s and homeodomain-interacting protein kinase-2 (HIPK2) are partners in regulating p53 activity. J. Biol. Chem..

[60] Shibuya H., Iinuma H., Shimada R., Horiuchi A., Watanabe T. (2011). Clinicopathological and prognostic value of microRNA-21 and microRNA-155 in colorectal cancer. Oncology.

[61] Frade R., Balbo M., Barel M. (2002). RB18A regulates p53-dependent apoptosis. Oncogene.

[62] Tu Y., Xie L., Chen L., Yuan Y., Qin B., Wang K., Zhu Q., Ji N., Zhu M., Guan H. (2020). Long non-coding RNA MEG3 promotes cataractogenesis by upregulating TP53INP1 expression in age-related cataract. Exp. Eye Res..

[63] Wang X., Wang L., Mo Q., Jia A., Dong Y., Wang G. (2016). A positive feedback loop of p53/miR-19/TP53INP1 modulates pancreatic cancer cell proliferation and apoptosis. Oncol. Rep..

[64] McCubrey J.A., Lertpiriyapong K., Fitzgerald T.L., Martelli A.M., Cocco L., Rakus D., Gizak A., Libra M., Cervello M., Montalto G., Yang L.V., Abrams S.L., Steelman L.S. (2017). Roles of TP53 in determining therapeutic sensitivity, growth, cellular senescence, invasion and metastasis. Adv. Biol. Regul..

[65] Kanehisa M., Sato Y., Kawashima M. (2022). KEGG mapping tools for uncovering hidden features in biological data. Protein Sci..

[66] Park S.J., Yoon B.H., Kim S.K., Kim S.Y. (2019). GENT2: an updated gene expression database for normal and tumor tissues. BMC Med. Genomics.

[67] Jin M., Park S.J., Kim S.W., Kim H.R., Hyun J.W., Lee J.H. (2017). Pig3 regulates p53 stability by suppressing its MDM2-mediated ubiquitination. Biomol. Ther..

[68] Kang M.Y., Kim H.B., Piao C., Lee K.H., Hyun J.W., Chang I.Y., You H.J. (2013). The critical role of catalase in prooxidant and antioxidant function of p53. Cell Death Differ..

[69] Tang Z., Li C., Kang B., Gao G., Li C., Zhang Z. (2017). GEPIA: a web server for cancer and normal gene expression profiling and interactive analyses. Nucleic Acids Res..

[70] Corre J., Hébraud B., Bourin P. (2013). Concise review: growth differentiation factor 15 in pathology: a clinical role?. Stem Cells Transl. Med..

[71] Zhou Y., Zhang X., Klibanski A. (2012). MEG3 noncoding RNA: a tumor suppressor. J. Mol. Endocrinol..

[72] Feroz W., Sheikh A.M.A. (2020). Exploring the multiple roles of guardian of the genome: P53, Egypt. J. Med. Hum. Genet..

[73] Li C., Tang Z., Zhang W., Ye Z., Liu F. (2021). GEPIA2021: integrating multiple deconvolution-based analysis into GEPIA. Nucleic Acids Res..

[74] Qian S., Wei Z., Yang W., Huang J., Yang Y., Wang J. (2022). The role of BCL-2 family proteins in regulating apoptosis and cancer therapy. Front. Oncol..

[75] Wei G.H., Wang X. (2017). lncRNA MEG3 inhibit proliferation and metastasis of gastric cancer via p53 signaling pathway. Eur. Rev. Med. Pharmacol. Sci..

[76] Xu J., Wang X., Zhu C., Wang K. (2022). A review of current evidence about lncRNA MEG3: a tumor suppressor in multiple cancers. Front. Cell Dev. Biol..

[77] Rama A.R., Hernandez R., Perazzoli G., Burgos M., Melguizo C., Vélez C., Prados J. (2015). Specific colon cancer cell cytotoxicity induced by bacteriophage E gene expression under transcriptional control of carcinoembryonic antigen promoter. Int. J. Mol. Sci..

[78] Rama Ballesteros A.R., Hernández R., Perazzoli G., Cabeza L., Melguizo C., Vélez C., Prados J. (2020). Specific driving of the suicide E gene by the CEA promoter enhances the effects of paclitaxel in lung cancer. Cancer Gene Ther..

[79] Xu C., Sun Y., Wang Y., Yan Y., Shi Z., Chen L., Lin H., Lü S., Zhu M., Su C., Li Z. (2012). CEA promoter-regulated oncolytic adenovirus-mediated Hsp70 expression in immune gene therapy for pancreatic cancer. Cancer Lett..

[80] Javan B., Shahbazi M. (2018). Constructing a novel hypoxia-inducible bidirectional shRNA expression vector for simultaneous gene silencing in colorectal cancer gene therapy. Cancer Biother. Radiopharm..

[81] Mayahi S., Golalipour M., Yamchi A., Jhingan G.D., Shahbazi M. (2019). New insights into the roles of the FOXO3 and P27Kip1 genes in signaling pathways, Ups. J. Med. Sci..

