# Supplementary material for: Harnessing the TP53INP1/TP53I3 axis for inhibition of colorectal cancer cell proliferation through MEG3 and Linc-ROR Co-expression
Source: Heliyon. 2024 Jul 10;10(14):e34075. doi: 10.1016/j.heliyon.2024.e34075 (PMC11301216; doi:10.1016/j.heliyon.2024.e34075)

***Harnessing the TP53INP1/TP53I3 Axis for Inhibition of Colorectal Cancer Cell Proliferation through MEG3 and Linc-ROR Co-expression***

Mahboobeh Ramezani^1, 3^, Fatemeh T. Shamsabadi^2, 3^, Majid Shahbazi^3, 4*^

1. Department of Genetics, Golestan University of Medical Sciences, Gorgan, Iran.
2. Department of Biotechnology, Golestan University of Medical Sciences, Gorgan, Iran. Fatima.shamsabadi@gmail.com
3. Medical Cellular & Molecular Research Center, Golestan University of Medical Sciences, Gorgan, Iran
4. AryaTinaGene (ATG) biopharmaceutical company, Gorgan, Iran

* Corresponding author:

Majid Shahbazi, Professor

Medical Cellular & Molecular Research Center, Golestan University of Medical Sciences, Gorgan, Iran.

Phone: 98-91215140251

E. mail: [shahbazimajid@yahoo.co.uk](mailto:shahbazimajid@yahoo.co.uk); [shahbazim@atgbio.com](mailto:shahbazim@atgbio.com)

**Keywords**: Colon cancer, MEG3, linc-ROR shRNA, p53

Supplementary Figures

1. Figure S1. (a) The schematic overview of the UM1(9577 bp), UM2 (7976 bp), and UM3 (9236 bp) vectors. The linc-ROR shRNA and MEG3 expression are regulated by a CEA promoter that is cloned into *Xho1* and *Xba1* sites. Vector design was performed using the SnapGene viewer software. (b) Agarose gel analysis for verification of UM1, UM2, and UM3. The UM1 was digested by *Sac* *I* and *ECOR* *I* restriction enzymes that separate a 2907 bp fragment (line 3). While lines 1 and 2 are undigested and linear vectors. Also, the UM2 and UM3 vectors were digested with *Xba I* (7976 bp) and *Xho I* (9236 bp) enzymes. Lines 1 and 3 are undigested UM2 and UM3 vectors. The DNA ladder (1Kb, ThermoFisher) was presented by M.


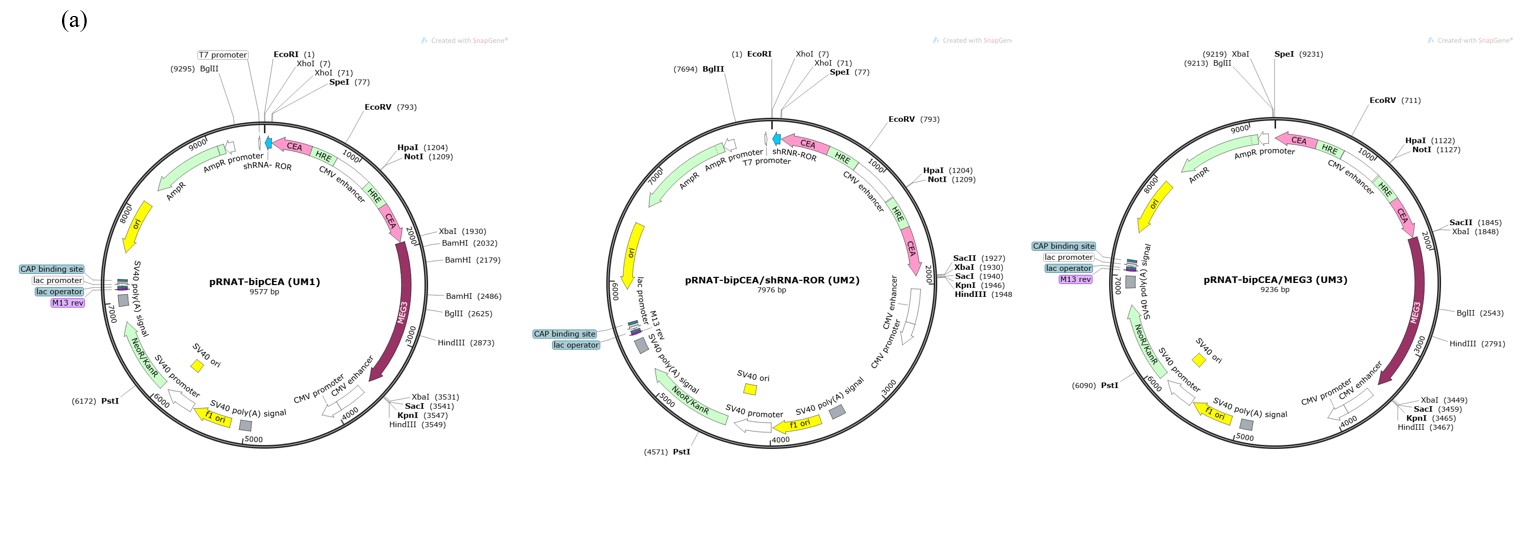


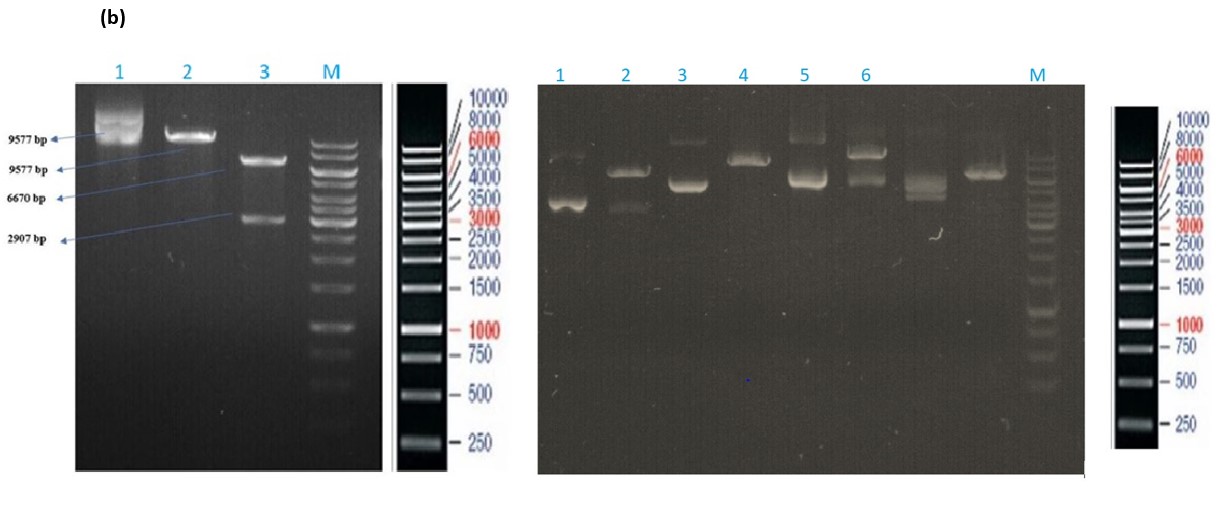

Supplement: Multimedia component 1 [file mmc1.docx]
